# Constitution of the Mississippi Valley Association of Dental Surgeons

**Published:** 1848-07

**Authors:** 


### CONSTITUTION

OF THE

### MISSISSIPPI VALLEY ASSOCIATION OF DENTAL SURGEONS.

### ARTICLE I.

This Society shall be called the Mississippi Valley Association of Dental Surgeons.

### ARTICLE II.

Sec. 1. The officers of this Society shall consist of a President, three Vice Presidents, a Recording, a Corresponding Secretary, Examining Committee, and Treasurer.

Sec. 2. The Officers shall be elected by ballot at each annual meeting, and their duties shall be such as appropriately belong to such officers in other Societies.

### ARTICLE III.

The Society now forming, shall be chosen from members of this convention by ballot, two-thirds of all the votes given being necessary to an election.

### ARTICLE IV.

After the formation of this Society, all applicants for membership shall be examined by the examining Committee, and in order to be received as members, they shall sustain a satisfactory examination on all the branches of Dental Science ; shall be twenty-one years of age, and give sufficient evidence of good moral character.

Those who have Diplomas from a respectable Dental Association, may be received without examination by the committee.

### ARTICLE V.

All candidates for membership, recommended by the Examining Committee, shall be balloted for, two-thirds of the votes given shall be necessary to membership.

### ARTICLE VI.

Any member may be expelled from the Society for immoral conduct, mal-practice, or other sufficient cause, on motion of an investigating committee appointed for that purpose, by a vote of two-thirds of all the members present.

### ARTICLE VII.

This Society shall be composed of two classes, viz.: acting and honorary members.

### ACTICLE VIII.

Each member, at his initiation, shall pay into the treasury of the Society, the sum of five dollars, and annually the sum of three dollars.*

* ARTICLE VIII—*As Amended.*

Each individual elected as acting member of this Society, shall within thirty days subscribe to the Constitution, and pay into the treasury of the Society five dollars, and annually thereafter the sum of three dollars; and all those who shall fail for two successive years to pay their annual dues, shall forfeit their membership.

### ARTICLE IX.

The meetings of this Society shall be held annually by adjournment from time to time and from place to place, agreeably to the will of the Society.

### ARTICLE X.

The Society may receive contributions in money, books or other property, which may be either used or sold in aid of its purposes.

### ARTICLE XI.

Seven acting members beside the President or Chairman, shall be necessary for the transaction of any business, at the opening of any annual meeting.

### ARTICLE XII.

In the event that a quorum cannot be obtained at any regular meeting of the Society, the time and place on the following year shall be the same as the preceding year. The President shall have the power in case of a failure of an annual meeting, to call a meeting of the Society at any time and place that may seem proper.

### ARTICLE XIII.

This Constitution may be amended by a vote of two-thirds of the members present at any annual meeting of the Society.

### OFFICERS FOR THE PRESENT YEAR.

Wm. B. Ross, *Pres.*
W. E. Ide, *1st Vice Pres.*
D. P. Hunt, *2d* " "
B. B. Brown, *3d* " "
J. J. Allen, *Recording Secretary.*
James Taylor, *Cor.* "
C. Bonsall, *Treasurer.*

W. H. Goddard, M. Rogers, E. Taylor, } *Examining Committee.*

Saml. Griffith, A. Berry, J. Allen, } *Executive* "

B. B. Brown, & Jas. Taylor, } *Editing and Publishing Com.*

The following gentlemen were appointed to deliver addresses at the next annual meeting—the President to deliver the opening address:

| | |
|---|---|
| Dr. B. B. Brown, | Subject not named. |
| " Wm. M. Hunter, | on Block Teeth. |
| " H. Crane, | " Plugging Teeth. |
| " Joseph Sanford | " Extracting Teeth. |
| " W. E. Ide, | " Irregularities of the Teeth. |
| " J. W. Cook, | " Cleanliness of the Mouth. |
| " C. Bonsall, | " Pivot Teeth. |

Society adjourned to meet on the 1st Tuesday of Sept., 1848, at 10 o'clock, A. M., in the Ohio College of Dental Surgery.

